# Supplementary material for: Learning curve and surgical outcome of robotic assisted colorectal surgery with ERAS program
Source: Sci Rep. 2022 Nov 29;12:20566. doi: 10.1038/s41598-022-24665-w (PMC9709162; doi:10.1038/s41598-022-24665-w)
Supplement: Supplementary file 1 — Supplementary Information 1. [file 41598_2022_24665_MOESM1_ESM.zip › LSC Raw data-Ñ[▒K/111/O_ñw╝╨░O▒Kñσ.pdf]

依據病歷管理會議決議，手術(前)摘要限[主治、主刀醫師]完成並設為正式！！
Tel:2508莫小姐

W52 / W52

臺中榮民總醫院

Operation Note

[redacted]

- 手術日期：2020/12/24
- 手術醫師：VS 蔣鋒帆 ，CR 陳映菁 ，Fellow 林昌霖 ，Fellow 黃侶霖 ，R 周奇霈 ，R 陳凱祥
- 執行麻醉：VS 蔡佩馨
- Anesthesia : GE
- Position : Lithotomy
- Aseptic agents : Soap , Betadine Alcohol , Betadine Aqua

Diagnosis

- Pre-operative diagnosis : Cecal tumor
- Post-operative diagnosis : Ditto.

Surgical Indication

- Tumor removal for pathologic proof

Operation

- 1. Laparoscopic right hemicolectomy with side-to-side anastomosis, GIA 80-3.8mm*2. re-enforced with 3-0 mersilk lambert suture.
  2. Vessel ligation: ileo-colic and right colic artery.
  3. Cut margin: proximal 8cm(terminal ileum), distal 8cm.
  4. A Fr.12 penrose drain placed at Morrison’s pouch.

Finding

- 1. A 1*5cm lateral spreading tumor at cecum, without invading to serosa layer, cT1-2N0M0
  2. There was no visible liver metastasis or visible peritoneal seeding.
  3. No synchronous polyp or tumor at resected specimen.

Procedure

- General endotracheal anesthesia was induced and then the patient was placed in the supine position with legs in adducted position. Preoperative antibiotics were given. A Foley catheter and nasogastric tube were placed. The abdomen was prepped and draped in standard sterile fashion. A small vertical incision was made above the umbilicus and a 12mm trocar was inserted into the peritoneal cavity by open method. Pneumoperitoneum was established with CO2 inflation. Laparoscope was inserted and then a 12mm trocar was inserted over LUQ of abdomen. Two 5mm trocars were inserted over RLQ and LLQ of abdomen. The liver, omentum and peritoneum were inspected for evidence of metastatic disease. No metastatic disease was noted. The small bowel was inspected and retracted to downward by grasper. The peritoneum overlying the mesentery was then scored with electrocautery and the ileocolic artery was identified, ligated with endoclips, and transected. The remaining vessels to the cecum were also divided and ligated. Using electrocautery, omentum was freed from the transverse colon and lesser sac was entered. The colon was freed from its peritoneal attachments along the line of Toldt from the cecum to the hepatic flexure. Hemostasis was checked in the operative field. Cecum, ascending colon and hepatic flexure were mobilized and then laparoscope was removed. A small midline incision was made over upper abdomen. The ascending colon was delivered into the incision and points of transection were selected proximally and distally. The bowel was divided. The two ends of the bowel were checked and found to be viable, with excellent blood supply. The fat was gently cleared from the terminal 2-3 mm of the bowel ends. The two ends of the bowel were brought into apposition and found to lie

- comfortably without excessive tension. GIA 80-3.8*2 was applied for side-to-side anastomosis, an outer layer of interrupted 3-0 silk Lambert sutures was used for re-enforcement. The anastomosis was checked and found to be intact and widely patent. Then the anastomosis was put back into peritoneal cavity, and pneumoperitoneum was created again. We checked bleeding meticulously and penrose drain was placed at Morrison pouch. The fascia of lower midline wound was closed with interrupted sutures of No.1 Vicryl. The fascia of the 12 mm trocar wound was closed with 1-0 vicryl. Skin were closed with interrupted 3-0 Nylon. The patient tolerated the procedure well and was brought to the POR unit in stable condition.
- Estimated blood loss: minimum
- 器械數量: 正確
- 紗布數量: 正確

Image

- PAINT
  - colon & rectum

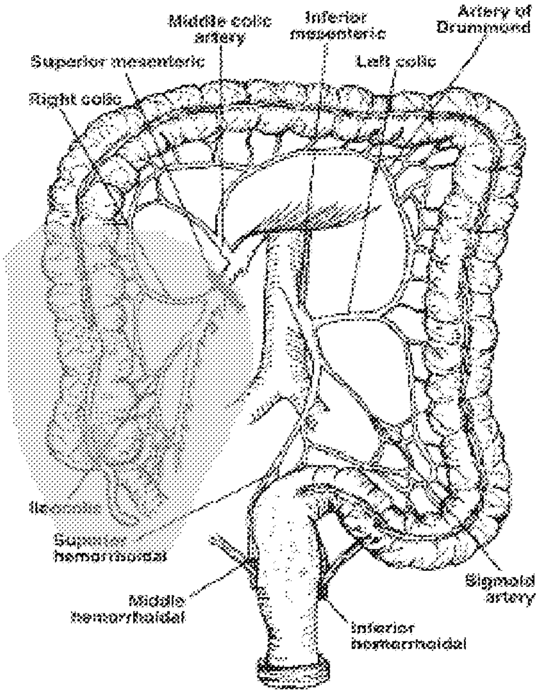

  - abdomen

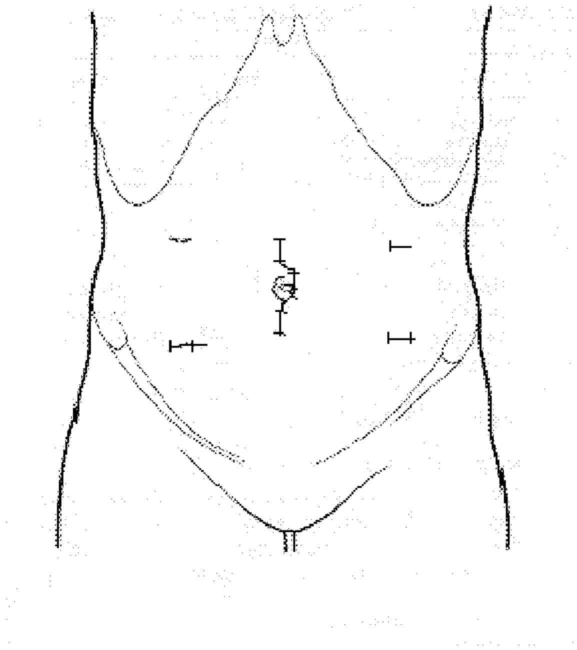

編輯醫師 2303K陳映菁

初步摘要

依據病歷管理會議決議，手術(前)摘要限[主治、主刀醫師]完成並設為正式！！ **Tel:2508** 莫小姐

W52 / W52

doctorRank:B
